# EGFR isoforms and gene regulation in human endometrial cancer cells

**DOI:** 10.1186/1476-4598-9-166

**Published:** 2010-06-25

**Authors:** Lina Albitar, Gavin Pickett, Marilee Morgan, Jason A Wilken, Nita J Maihle, Kimberly K Leslie

**Affiliations:** 1 The Reproductive Molecular Biology Laboratory, Division of Maternal-Fetal Medicine, Departments of Obstetrics and Gynecology, Biochemistry and Molecular Biology, and Biomedical Sciences, University of New Mexico Health Sciences Center, 2211 Lomas Boulevard Northeast, MSC10 5580, Albuquerque, NM 87131, USA; 2 KUGR Microarray and Genomics Facility, University of New Mexico Health Sciences Center, 2325 Camino de Salud NE, Albuquerque, NM 87131, USA; 3 Department of Obstetrics, Gynecology, & Reproductive Sciences, Yale University School of Medicine, PO Box 208063, 310 Cedar St., New Haven, CT 06520, USA; 4 Departments of Pathology, and Pharmacology, Yale University School of Medicine, PO Box 208063, 310 Cedar St., New Haven, CT 06520, USA; 5 Department of Obstetrics and Gynecology, Harvard Medical School, Brigham and Women's Hospital, 75 Francis St., Boston, MA 02115, USA; 6 Mind Research Network, 1101 Yale Boulevard NE, Albuquerque, NM 87106, USA; 7 Department of Obstetrics and Gynecology, University of Iowa. Hospitals and Clinics, 200 Hawkins Drive, Iowa City, IA 52242, USA

## Abstract

**Background:**

Epidermal growth factor (EGF) and its receptor (EGFR) constitute a principal growth-promoting pathway in endometrial cancer cells. Pre-clinical studies were undertaken to compare the expression of EGFR isoforms and the downstream effects of activating or blocking EGFR function in Ishikawa H cells, derived from a moderately differentiated type I endometrioid adenocarcinoma, or in Hec50co cells, derived from a poorly differentiated type II adenocarcinoma with papillary serous sub-differentiation.

**Results:**

We investigated whether EGFR mutations are present in the tyrosine kinase domain (exons 18-22) of EGFR and also whether EGFR isoforms are expressed in the Ishikawa H or Hec50co cell lines. Sequence of the EGFR tyrosine kinase domain proved to be wild type in both cell lines. While both cell lines expressed full-length EGFR (isoform A), EGFR and sEGFR (isoform D) were expressed at significantly lower levels in Hec50co cells compared to Ishikawa H cells. Analysis of gene expression following EGF vs. gefitinib treatment (a small molecule EGFR tyrosine kinase inhibitor) was performed. Early growth response 1, sphingosine kinase 2, dual specificity phosphatase 6, and glucocorticoid receptor DNA binding factor 1 are members of a cluster of genes downstream of EGFR that are differentially regulated by treatment with EGF compared to gefitinib in Ishikawa H cells, but not in Hec50co cells.

**Conclusions:**

Type I Ishikawa H and type II Hec50co endometrial carcinoma cells both express EGFR and sEGFR, but differ markedly in their responsiveness to the EGFR inhibitor gefitinib. This difference is paralleled by differences in the expression of sEGFR and EGFR, as well as in their transcriptional response following treatment with either EGF or gefitinib. The small cluster of differently regulated genes reported here in these type I vs. type II endometrial cancer-derived cell lines may identify candidate biomarkers useful for predicting sensitivity to EGFR blockade.

## Background

Endometrial carcinoma is the most common gynecologic malignancy in American women [1-3]. Type I endometrial cancers are generally of endometrioid subtype, well differentiated, express estrogen and progesterone receptors (ER and PR), and develop in a setting of estrogen excess unopposed by the differentiating effects of progesterone [4,5]. Ishikawa H, a cell line derived from a moderately differentiated endometrioid type I adenocarcinoma [6], is hormone receptor positive and forms rudimentary glandular structures in culture [7,8]. In contrast, type II endometrial cancers include clear cell, serous, and poorly differentiated endometrioid subtypes, are poorly differentiated and result in more aggressive lesions [4,5]. Type II tumors are typically resistant to hormonal growth regulation because they express less ER and PR. Hec50co cells were derived from a metastatic type II endometrial cancer and sub-differentiate into a serous subtype in xenografted animal models [9]. Hec50co cells are poorly differentiated in culture and do not express appreciable levels of ER or PR [6].

No effective treatment is available for persistent or recurrent endometrial cancer. New therapies using the rationale that cancer cells express or amplify certain signaling proteins, such as the epidermal growth factor receptor (EGFR) family of tyrosine kinase receptors, are under investigation, as described below.

EGFR is the prototypic member of the ErbB/HER receptor tyrosine kinase family and binds to multiple ligands including EGF, transforming growth factor alpha, and amphiregulin. EGFR plays a crucial role in cellular functions implicated in cancer development [10], and has been shown to be expressed in a large percentage of endometrial tumors [11]. We previously investigated the expression of EGFR and identified its downstream signaling cascades in both Ishikawa H and Hec50co cells [12]. Tyrosine kinase inhibitors block EGFR autophosphorylation in both cell lines *in vitro *[12]. However, the well-differentiated Ishikawa H cell line responds more robustly to EGFR activation and is more sensitive to receptor inhibition compared to Hec50co cells, which are relatively resistant. Specifically, fewer signaling intermediates are activated or blocked downstream of EGFR in Hec50co cells compared to Ishikawa H cells [12]. Also, cell cycle regulatory events in response to the EGFR tyrosine kinase inhibitor gefitinib are blunted in Hec50co cells compared to Ishikawa H cells [13]. The reason these poorly differentiated cells do not respond as well to inhibition of EGFR activity is an interesting question that may have bearing on resistance to tyrosine kinase inhibitors in the clinical setting.

**Figure 1:**
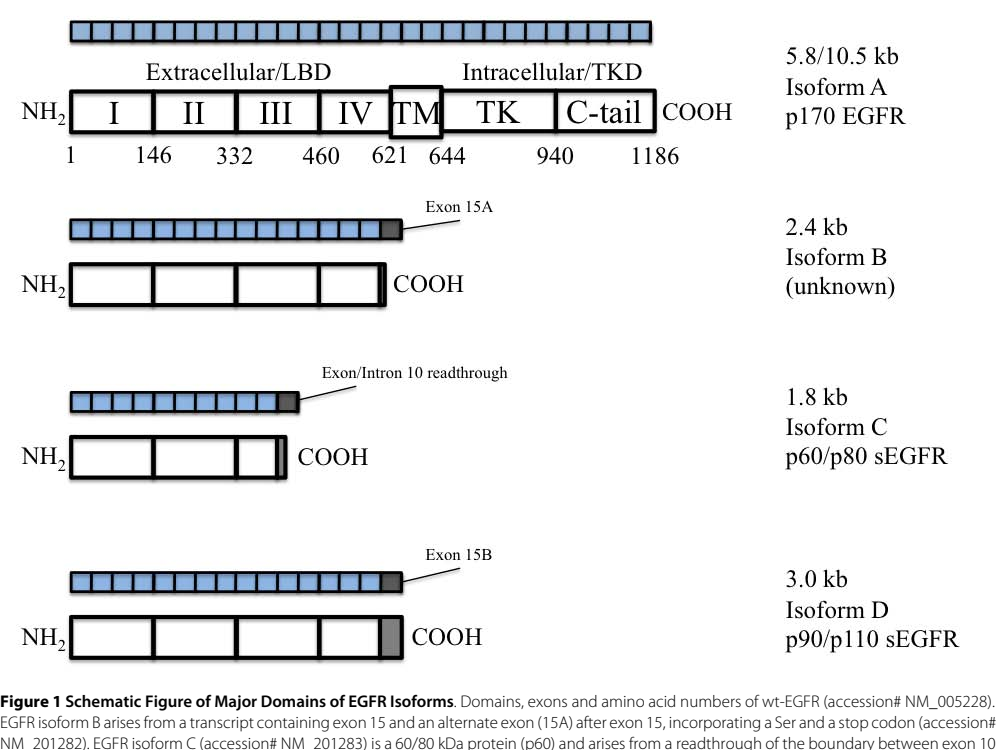
**Schematic Figure of Major Domains of EGFR Isoforms**. Domains, exons and amino acid numbers of wt-EGFR (accession# NM_005228). EGFR isoform B arises from a transcript containing exon 15 and an alternate exon (15A) after exon 15, incorporating a Ser and a stop codon (accession# NM_201282). EGFR isoform C (accession# NM_201283) is a 60/80 kDa protein (p60) and arises from a readthrough of the boundary between exon 10 and intron 10-11, incorporating 2 unique carboxy-terminal amino acids (Leu-Ser) and a stop codon. EGFR isoform D (accession# NM_201284) is a 90/110 kDa protein (p110/sEGFR) and arises from incorporation of an alternate exon (15B) after exon 15, incorporating 78 unique amino acids and a stop codon.

Human EGFR is encoded by two transcripts of 10.5 kb and 5.8 kb (isoform A) both of which arise from a single promoter region/gene on chromosome 7 [14]; the protein product arising from these two transcripts is identical. In addition to these two transcripts which encode the full-length EGFR isoform, three alternative transcripts of 1.8, 2.4, and 3.0 kb, also are derived from the *EGFR *gene and encode isoforms C, B, and D, respectively [15,16]. While the 1.8 kb transcript results from read-through of an exon (10) intron boundary, the 2.4 and 3.0 kb transcripts, encoding isoforms B and D transcripts diverge from full-length EGFR by incorporating alternate exons 15A or 15B. Exon 15A encodes a unique carboxy-terminal serine; exon 15B encodes an alternative 78-amino acid C-terminal sequence (isoform D). Both of these alternately spliced transcripts also encode alternative stop codons as well as unique 3' untranslated regions (UTR) including consensus polyadenylation sites (see Figure 1). While the 2.4 kb isoform B and its protein product have not been well studied, the 3.0 kb isoform D transcript encodes a 90/110 kDa EGFR isoform that is associated with the cell membrane through an unidentified mechanism [17] and also can be detected in human serum [18,19]. These soluble receptor isoforms resemble the avian secreted sEGFR isoform, which has been shown to bind to ligand and to inhibit ligand-dependent, anchorage-independent growth of primary fibroblasts [15,20]. sEGFR/sHER receptors also have been reported to modulate EGFR/HER tyrosine kinase activity [21,22]. The 1.8 kb isoform C transcript codes for a secreted 60/80 kDa soluble EGFR protein that contains only subdomains I, II, and half of subdomain III of the EGFR extracellular region followed by a unique carboxy-terminal Leu-Ser and 3' UTR (Figure 1).

The tyrosine kinase inhibitor gefitinib (Iressa, ZD1839) binds to the ATP binding site of the EGFR kinase domain with a higher affinity than does ATP itself [23]. Heterozygous somatic mutations of the EGFR tyrosine kinase domain (exons 18-21) have been correlated with a positive response to gefitinib [24,25]. These missense (G719S/C and L858R) and deletion mutations (in the region spanning codons 746-759) are located in exons 18 through 21 of EGFR and appear to confer tumor susceptibility to gefitinib [24,25]. It has been hypothesized that such mutations stabilize the interaction of EGFR with ATP or gefitinib thereby resulting in an increase in both ligand activation and inhibitor de-activation [24]. These mutations are distinct from the variant EGFRvIII, discovered in glioma and metastatic breast cancer, where mutant receptors maintain constitutively active, ligand-independent receptor activity; moreover, these latter extracellular domain EGFR mutants do not interfere with the ability of the receptor tyrosine kinase domain to bind to gefitinib [24].

Here, we have sequenced the tyrosine kinase domain (exon 18-22) of EGFR and also have determined the expression of these EGFR isoforms in the two endometrial carcinoma cell lines described above. In addition, we have evaluated the effects of gefitinib or EGF on gene expression to better understand how these two cell lines respond to treatment. We hypothesized that Ishikawa H cells, previously found to be more responsive to EGFR modulation with respect to signaling and cell cycle inhibition, would express functional EGFR and show a robust transcriptional response to EGF [12,13]. By contrast, we hypothesized that Hec50co cells, previously found to be more resistant to EGFR modulation with respect to signaling [12] and cell cycle inhibition [13], might differentially express EGFR or its related isoforms, and that these expression patterns may predict a blunted transcriptional response to EGF as well as to gefitinib. The results of these pre-clinical studies contribute to our understanding of the distinguishing cellular characteristics that control drug performance in two well-characterized models of endometrial cancer.

## Methods

### Drugs and chemicals

EGF was purchased from Invitrogen (Carlsbad, CA). Gefitinib (Iressa, ZD1839) was provided by AstraZeneca (Wilmington, DE and Cheshire, UK). The drug was dissolved in dimethyl sulfoxide (DMSO) for all *in vitro *studies.

### Cells and culture conditions

Ishikawa H and Hec50co cells were provided by Dr. E. Gurpide, New York University, and were cultured in DMEM media (Sigma-Aldrich Inc., St. Louis, MO) supplemented with 10% fetal bovine serum substitute (FetalPlex, Gemini Bio-Products, Woodland, CA), 2 mM L-Glutamine and 1× antibiotic-antimycotic solution (GIBCO, Grand Island, NY). Cells were incubated either with 0.1% DMSO (vehicle), 1 μM of gefitinib, or with 30 ng/ml EGF for 12 and 24 h before harvesting for gene array or immunoblot studies as described below.

### RNA isolation and microarray hybridization

Ishikawa H and Hec50co cells were cultured and treated as described above. Cells were harvested by scraping, and the pellets were washed twice with phosphate buffered saline (PBS). Total RNA was prepared from the cell pellets according to the manufacturer's protocol using RNeasy spin columns (Qiagen Corp, Valencia, CA). RNA quality was checked using the Agilent 2100 Bioanalyzer (Agilent Technologies, Foster City, CA). All microarray procedures were performed using the human Affymetrix™ HG-U133 plus 2.0 chips (Santa Clara, CA). Procedures for the chip preparation and cDNA/cRNA synthesis were performed according to instructions from the manufacturer's manual, version 701025 Rev.5. Briefly, 5 μg of total RNA was used to generate double-stranded cDNA using an oligo dT-primer containing the T7 RNA polymerase promoter site and the One-Cycle Target Labeling Kit. cDNA was purified via column purification using the GeneChip Sample Cleanup Module, and biotinylated cRNA was synthesized by *in vitro *transcription using the GeneChip IVT Labeling kit. Biotin labeled cRNA was purified (GeneChip Sample Cleanup Module), and the absorbance was measured at 260 nm to determine yield (Nanodrop spectrophotometer). Twenty μg of the labeled cRNA was fragmented; the quality of the purified cRNA and the fragmented cRNA was assessed using the Agilent 2100 Bioanalyzer and the RNA 6000 Nano LabChip kit. The labeled fragmented cRNA was hybridized to Affymetrix GeneChipHGU133 Plus 2.0 arrays for 16 h at 45°C following the Affymetrix protocol specific to this array type. The washing and staining steps were performed on the Affymetrix 450 fluidics station according to the antibody amplification protocol (Fluidics script: EukGE-WS2v5). The GeneChips were scanned using the Affymetrix GeneChip Scanner 3000 (a wide-field, epifluorescent near-confocal microscope with a patented flying objective).

### Real time PCR (qPCR)

Synthesis of cDNA from total RNA was performed using the High-Capacity cDNA Archive Kit from Applied Biosystems, part number 4322171 (Foster City, CA). One mg of total RNA was converted to cDNA in a μ1 ml 10× Reverse Transcription Buffer, 0.4 μl 25× dNTPs, μ1.0 ml 10× random primers, 0.5 μl MultiScribe™ reverse transcriptase, 50 U/μl and 7.1 ml nuclease-free water. The reactions were incubated for 10 min at 25°C followed by 120 min at 37°C. Singleplex real time PCR was performed in 384 well format using the Applied Biosystems 7900 HT. Reactions were prepared in 10 ml volumes containing 10 ng cDNA, 0.5 ml of the 20× Target Assay Mix or endogenous control, and 5.0 μl 2× TaqMan Universal Master Mix. Relative quantification was obtained using the Comparative C_t _method. This involves comparing the C_t _values of the samples of interest with a control or calibrator such as a non-treated sample or RNA from normal tissue. We used untreated samples as calibrators. The C_t _values of both the calibrator and the samples of interest were normalized to an appropriate endogenous housekeeping gene (18S).

### Reverse transcriptase polymerase chain reaction (RT-PCR)

First strand synthesis was performed according to the instructions provided by Invitrogen™ Life Technologies (Carlsbad, CA). For these studies, 1.0 μg of total RNA was used to generate single-stranded cDNA via first strand synthesis using SuperScript™ III in the presence of oligo-(dT)_20 _primer and dNTP's. The mixture was heated to 65°C for 5 min and placed on ice for at least 1 min. First Strand Buffer, dithiothreitol and SuperScript III were added per the protocol directions and incubated for 1 h at 55°C followed by inactivation by heating at 70°C for 15 min. PCR was performed according to the instructions provided by Qiagen, Inc (Valencia, CA) for their HotStarTaq Master Mix product. A total of 100 ng of cDNA was added to the reaction as a template along with the appropriate primers and the PCR master mix. An annealing temperature of 59°C was used, and PCR was performed over 35 cycles. Equal amounts of the products were separated by agarose gel electrophoresis, and a size marker was included for molecular weight determination.

### Immunoblot analysis of EGFR isoforms

Near-confluent cultures of Ishikawa H and Hec50co cells were rinsed 3× with ice-cold PBS and harvested by scraping. Cells were solubilized with boiling SDS-lysis solution (2.5% SDS, 0.5% NP-40, 0.5% deoxycholate) for five minutes. Following solubilization, lysates were diluted 1:9 with 50 mM Tris-HCl, pH 7.4, 190 mM NaCl, 6 mM EDTA, 2.5% Triton X-100, and protease inhibitors (2 mM PMSF, 1 mg/ml aprotinin, pepstatin, and leupeptin). Cell lysate protein concentration was quantitated by Bio-Rad DC assay.

CHO/EGFR and CHO/sEGFR [26] lysates were used as positive controls for EGFR and sEGFR expression, respectively. Samples were loaded onto 7.5% acrylamide, 1.5 mm-thick Hoeffer slab gels and electrophoresed for 4 hours at 30 mA (along with Bio-Rad Precision Plus MW markers), followed by transfer to PVDF membrane by semi-dry electroblotting (418 mA for 30 min). The membrane was blocked for one hour with 5% non-fat dry milk in 10 mM Tris-HCl, pH 7.4, 150 mM NaCl (TBS), and rinsed 6× for 5 min with wash buffer (TBS plus 0.1% Tween-20). The membrane was then successively probed with antibody directed against β-tubulin (Cell Signaling Technology, Danvers, MA, 1:500) and EGFR (sc-03, Santa Cruz Biotechnology, Santa Cruz, CA, 1:500) or sEGFR (1:4000) [26] in TBS plus 0.1% bovine serum albumin (BSA) overnight, followed by 6× rinses with wash buffer for 10 min each. The sEGFR-specific antibody is directed against the unique carboxy-terminal sequence of the 3.0 kb sEGFR gene product [27], whereas the sc-03 anti-EGFR antibody is directed against the EGFR intracellular domain. Membranes were then incubated with secondary antibody (goat anti-rabbit, 1:4000, Pierce) in TBS with 0.1% BSA for one hour followed by 6× rinses with wash buffer for 10 min each. Immune complexes were detected by enhanced chemiluminescence (Supersignal West Femto, Pierce).

### Sequencing exons 18-22 of EGFR

**Figure 2:**
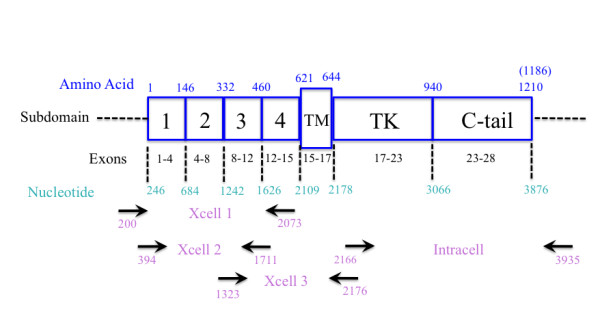
**Schematic Figure of Wild Type EGFR Domains and Subdomains**. This figure shows the number of exons, amino acids, and nucleotides in each domain. Arrows correspond to selected primers used to identify wild type EGFR and EGFR isoforms. Numbers below the arrows match the location of the primers on EGFR RNA sequence (accession#NM_005228). Names between arrow pairs correspond to the name of the primer set; Xcell and Intracell specify sequences encoding the extracellular and intracellular domains of EGFR (see Table 1).

cDNA was generated as described above using primers to query each EGFR subdomain (Figure 2). PCR was performed to generate templates for sequencing (standard PCR protocol, T_m _of 59°C). Samples were sequenced at the DNA Research Services Core Laboratory of the University of New Mexico (UNM) using an ABI 377 genetic analyzer, capillary electrophoresis and ABI big dye terminator ready reaction kits (Applied Biosystems, Carlsbad, CA). The automated instrument system performs electrophoretic separation and spectral detection of dye-labeled DNA fragments to determine base sequence, fragment size, or relative quantity. Sequencing reactions are performed with fluorescent labels: four different dyes identify the A, C, G, and T extension reactions. Primers used for sequencing EGFR (exon 18-22) are presented in Table 1.

**Table 1:** Primer Sets for Amplification of EGFR Isoforms and Mutants.

Primer set name	Sequence	Expected band size (bp)
EGFR forward extracellular 1 (Xcell 1)	ccagtattgatcgggagagc	1873
	
EGFR reverse extracellular 1 (Xcell 1)	acaacaccctggtctggaag	

EGFR forward extracellular 2 (Xcell 2)	agcctccagaggatgttcaa	1318
	
EGFR reverse extracellular 2 (Xcell 2)	tggttttctgaccggaggt	

EGFR forward extracellular 3 (Xcell 3)	caaaaactgcacctccatca	854
	
EGFR reverse extracellular 3 (Xcell 3)	ggatcttaggccccattcgtt	

EGFR forward intracellular (Intracell)	cctaagatcccgtccatcg	1769
	
EGFR reverse intracellular (Intracell)	ttggtcctgggtatcgaaag	

EGFR isoform B forward	aacaacaccctggtctggaa	160
	
EGFR isoform B reverse	tgaagcaaagggagaaattga	

EGFR isoform C forward	ggatattctgaaaaccgtaaaggaaa	96
	
EGFR isoform C reverse	cgaaaagttctctctaaaacactgatt	

EGFR isoform D forward	ccagtgtgcccactacattg	221
	
EGFR isoform D reverse	cgctgccatcattactttga	

Sequencing primers (exon 18-22) forward	ccaaccaagctctcttgagg	540
	
Sequencing primers (exon 18-22) reverse	tgataggcactttgcctcct	

### Analytical approaches and data interpretation

We analyzed the effects of EGF and gefitinib on gene expression in Ishikawa H and Hec50co cells at 12 h and 24 h normalized against specific controls. Two separate experiments were run for each cell line, treatment, and time point. Silicon Genetics' GeneSpring version 7.2 (Palo Alto, CA) was used to filter data using a fluorescent-threshold cutoff and a 2-fold differential cutoff. Two different normalizations were employed: median-normalization and normalization to specific samples, depending on the comparisons being examined. Data from the two cell lines, Ishikawa H and Hec50co, were further compared using Venn diagrams. In addition, we employed Ingenuity™ software (Redwood City, CA) to generate common networks between linked genes with analytical changes of 2-fold or more [28].

## Results

### EGFR sequencing in Ishikawa H and Hec50co cells

**Figure 3:**
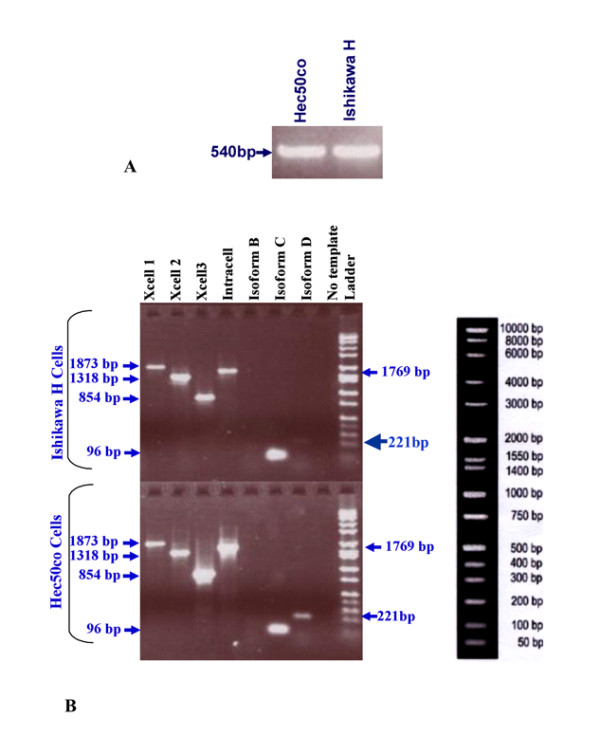
**RT-PCR Agarose Gel of EGFR cDNA in Ishikawa H and Hec50co Cells**. A) Band corresponds to EGFR exons 18-22, 540 bp in size, in Hec50co and Ishikawa H cells. B) Bands correspond to extracellular and intracellular domains of EGFR well as sEGFR isoforms B, C, and D.

Exons 18-22 of the tyrosine domain of EGFR of Ishikawa H and Hec50co cells were sequenced (Figure 3). PCR products were detected on a DNA agarose gel (Figure 3A) and were found to be the expected size (540 bp). No mutations in the tyrosine kinase domain of EGFR (exons 18-22) were observed in either cell line at the amino acid level. Two conservative single nucleotide polymorphisms were identified that did not change the amino acid sequence (data not shown); therefore, we conclude that the EGFR tyrosine kinase domains are wild type in both cell lines.

### EGFR isoforms in Ishikawa H and Hec50co cells

**Figure 4:**
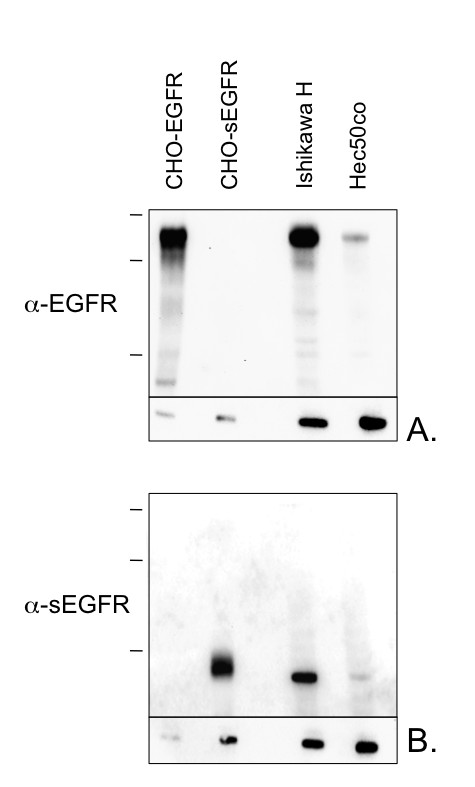
**Immunoblot Analysis of EGFR Isoform Expression in Ishikawa H and Hec50co Cells**. Cell lysates from endometrial or transfected control (CHO-EGFR, CHO-sEGFR) cell lines were resolved by SDS-PAGE (7.5% acrylamide), and immunoblotted using enhanced chemiluminescence with antibodies specific for either: A) EGFR (isoform A, p170, intracellular domain), or B) sEGFR (isoform D, p90/110). Anti-tubulin labelling is included as a loading control (lower panel, Figs. A and B). Molecular weight markers migrate at the positions indicated: 250 kD, 150 kD, and 100 kD.

Detection of EGFR isoforms in Ishikawa H and Hec50co cells was conducted initially by RT-PCR. We designed multiple primer sets specific for the EGFR extracellular and intracellular domains (Figure 2). In addition, primers that span regions of the internally-deleted variant and the soluble isoforms were included (Table 1; see Materials and Methods). The products of these reactions (Figure 3B) were expected to identify the expression of EGFR wild type and soluble isoforms (Figure 1) as well as EGFRvIII. PCR products were analyzed by agarose gel electrophoresis to confirm size and abundance, using primer templates as negative controls (Figure 3B). As determined from the primer set locations and band sizes on agarose gels, EGFRvIII expression was not detected. Using semi-quantitative methods, we also detected the message for the 1.8 kb sEGFR isoform (isoform C) at comparable levels in both Ishikawa H and Hec50co cells (Figure 3B). By these same methods the 3.0 kb sEGFR transcript (isoform D, Figure 3B) appeared to be expressed at higher levels in the Hec50co cells relative to the Ishikawa H cells (isoform D transcript is just detectable in Ishikawa cells). The 2.4 kb isoform (B) was not observed in either cell line. However, RT-PCR is not quantitative, and when immunoblot analysis was performed to assess isoform D protein expression (Figure 4), more sEGFR protein was detected in Ishikawa H cells using an antibody specific for the unique carboxy-terminal sequence of isoform D [26] than was detected in Hec50co cells. Both cell lines expressed wild type EGFR with higher levels in Ishikawa H cells (Figure 4).

### Effects of EGF and gefitinib on gene expression in Ishikawa H cells

Ishikawa H cells were incubated with either 30 ng/ml EGF or 1 μM gefitinib for 12 and 24 h. Cells were harvested, and total RNA was isolated for microarray studies using human Affymetrix™ HG-U133 plus 2.0 chips. The expression of 134 and 150 genes was altered 2-fold or more at 12 and 24 h, respectively, after exposure to EGF. Forty-four genes were regulated both at the 12 and 24 h time points (Additional File 1, Table S1). The expression of 132 and 61 genes was altered at 12 and 24 h, respectively, after gefitinib treatment. Twenty-five genes were found to be regulated at both the 12 and 24 h time points (Additional File 1, Table S1). While most genes were induced or down-regulated similarly at 12 and 24 h, some genes were differentially regulated. For example, the expression of Splicing Factor 4 (SF4) was decreased by 0.472 fold at 12 h and increased by 3.639 fold at 24 h in response to EGF in Ishikawa H cells. Sphingosine kinase 2 (SphK2) and protein inhibitor of activated STAT (PIASy) showed a similar pattern in response to gefitinib: decreased at 12 h but increased at 24 h. For those genes regulated by both EGF and gefitinib, the majority demonstrated an induction by one treatment and a down-regulation by the other, indicating that activation and blockade of EGFR resulted in predictably opposing effects on gene expression (Additional File 1, Table S1). For example, early growth response 1 (EGR1), epithelial membrane protein 1, FOS-like antigen 1, dual specificity phosphatase 6 (DUSP6), plasminogen activator, urokinase, sprouty homolog 2, sprouty homolog 4, and potassium voltage-gated channel, Isk-related family member 3 (KCNE3) were all induced by EGF and down-regulated by gefitinib at 12 h. Epithelial membrane protein 1, DUSP6, EGR1, sprouty homolog 2, sciellin, 5'-nucleotidases, ecto (CD73), and KCNE3 were all induced by EGF and down-regulated by gefitinib at 24 h.

### Effects of EGF and gefitinib on gene expression in Hec50co cells

Hec50co cells were incubated with either 30 ng/ml EGF or 1 μM gefitinib for 12 and 24 h. Cells were harvested, and total RNA was isolated as above. After excluding the hypothetical gene products and the ESTs, the expression of 35 and 58 genes was altered in response to EGF at 12 and 24 h compared to untreated control cells. Three genes (fibroblast growth factor receptor 2, stanniocalcin 1, and kinesin family member 26A) were found to be regulated at both the 12 and 24 h time points in response to EGF. The expression of 14 and 59 genes was altered in response to gefitinib after 12 and 24 h, respectively, in comparison to untreated controls. One gene, calcium homeostasis endoplasmic reticulum protein, was found to be regulated at both 12 and 24 h (Additional File 1, Table S1).

### Effects of EGF and gefitinib on gene expression in Ishikawa H vs. Hec50co cells

Gene expression in response to EGF and gefitinib was further analyzed to determine commonly regulated genes. Two genes, EGR1 (up-regulated) and polycystic kidney disease 1-like (down-regulated), were commonly regulated in both Ishikawa H and Hec50co cells in response to EGF at 12 h (Additional File 1, Table S1). Three down-regulated genes (papilin-proteoglycan-like sulfated glycoprotein, fibroblast growth factor receptor 2, and cadherin 16, KSP-cadherin) were similarly regulated in both cell lines in response to EGF at 24 h. One gene, glucocorticoid receptor DNA binding factor (GRLF1), was found to be differentially controlled by gefitinib (down-regulated in Ishikawa H and up-regulated in Hec50co cells) at 12 h. SphK2 and PIASy were two common genes (both induced) in Ishikawa H and Hec50co cells after 24 h of gefitinib treatment based on these gene array data.

### Real time PCR confirms changes in gene expression for selected genes

**Figure 5:**
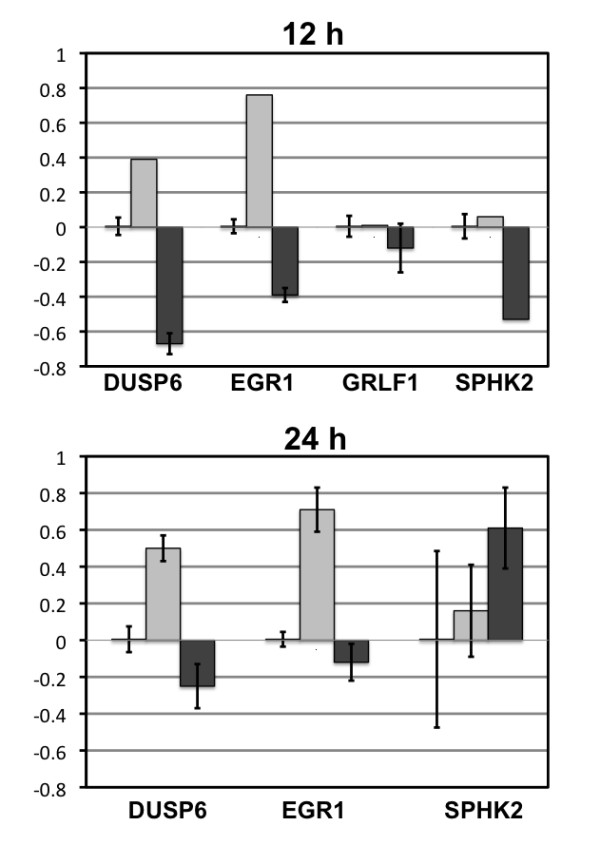
**Real Time PCR Graphs for Chosen Genes in Ishikawa H cells**. Data from qPCR confirmed the change in expression from the arrays for genes DUSP6 (dual specificity phosphatase 6), EGR1 (early growth response 1), GRLF1 (glucocorticoid receptor DNA binding factor), and SPHK2 (sphingosine kinase 2) in response to EGF and gefitinib at 12 and 24 h. Calibration was to the corresponding control at the specific time points.

**Figure 6:**
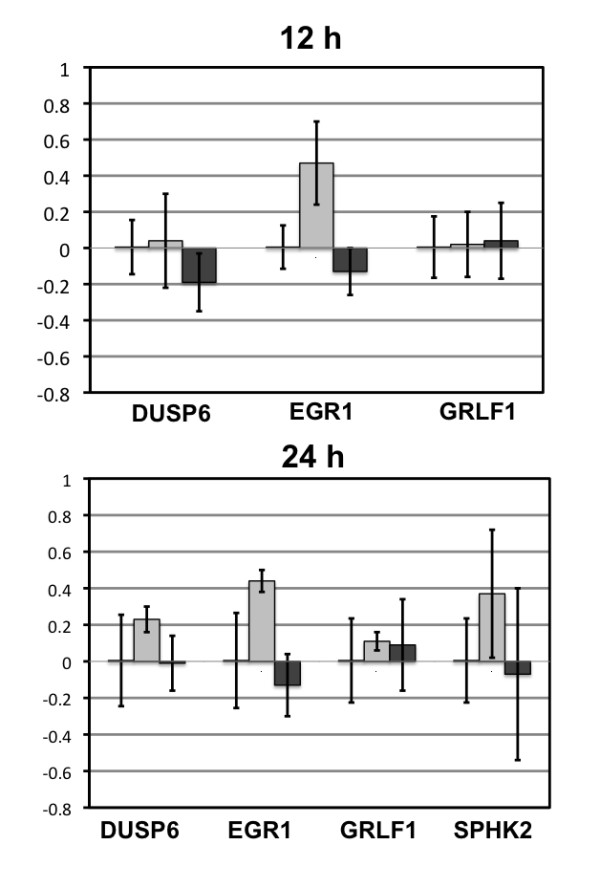
**Real Time PCR Graphs for Chosen Genes in Hec50co Cells**. from qPCR confirmed the change in expression from the arrays for genes DUSP6 (dual specificity phosphatase 6), EGR1 (early growth response 1), GRLF1 (glucocorticoid receptor DNA binding factor), and SPHK2 (sphingosine kinase 2) in response to EGF and gefitinib at 12 and 24 h. Calibration was to the corresponding control at the specific time points.

Findings from the array experiments were confirmed for four genes by qPCR in Ishikawa H cells (Figure 5) and Hec50co cells (Figure 6). These four transcripts were chosen from diverse functional categories. We were interested in common genes between Ishikawa H and Hec50co cells that would comprise a core set of transcripts downstream of EGF/EGFR. We identified two genes in this category for confirmation: EGR1, that was induced by EGF at 12 h (3.365 fold in Ishikawa H and 2.442 fold in Hec50co cells); and glucocorticoid receptor DNA binding factor 1 (GRLF1), that was down-regulated by gefitinib at 12 h in Ishikawa H cells (0.483 fold) and induced in Hec50co cells (2.73 fold). In addition, SphK2 was of special interest because it was differentially expressed in response to gefitinib over time in Ishikawa H cells (down-regulated at 12 h and induced at 24 h). DUSP6 is also a gene downstream of EGFR signaling pathways. DUSP6 was induced by EGF and down-regulated by gefitinib treatment at 12 h and 24 h in Ishikawa H cells (Additional File 1, Table S1).

### Gene networks

Analyzing the Affymetrix expression array data using Venn diagrams and Ingenuity™ networks provided additional insight into the transcriptional response of Ishikawa H and Hec50co cells to EGF and gefitinib. Ishikawa H cells are clearly more responsive to gefitinib, but Hec50co cells demonstrate similarities as well as interesting differences, including the induction of a group of pro-proliferative and compensatory factors at 24 h. Such factors may be markers of resistance to gefitinib worthy of future validation. These data are provided in the Additional Files of this manuscript and discussed below.

## Discussion

To evaluate the potential therapeutic effects of tyrosine kinase inhibitors in the treatment of endometrial cancer, we previously have characterized the EGFR pathway in endometrial cancer cells at the signaling level [12], with respect to cell cycle [13], and now at the genomic level. First we sequenced the tyrosine kinase domain of EGFR (exon 18-22) and identified the isoforms present in Ishikawa H cells (moderately differentiated) and Hec50co cells (poorly differentiated). In both cell types, no mutations were detected in EGFR that modify the amino acid sequence in the ATP binding pocket. Transcripts encoding the alternate EGFR isoforms, C and D, were detected in both cell lines. Immunoblot analysis with an antibody specific for sEGFR isoform D demonstrated that Ishikawa H cells express more isoform D compared to Hec50co cells (Figure 4). It is unclear whether this finding relates to the relative sensitivity of Ishikawa H cells to gefitinib compared to more resistant Hec50co cells. However, decreased serum levels of this same sEGFR isoform have been reported in both ovarian [18,29] as well as endometrial cancer patients (preliminary findings, Leslie et al., unpublished results, and ASCO, 2009), and higher serum sEGFR concentrations have been correlated with responsiveness to gefitinib in both lung and colon cancer patients [30,31]. Since serum sEGFR arises from the isoform D transcript, there may be a link between these observations, and further study of the function of this sEGFR isoform in endometrial cancer is clearly warranted. In this regard, serum sEGFR concentrations also have been correlated with the female gonadotropin follicle stimulating hormone, as well as with responsiveness to treatment with the aromatase inhibitor letrozole in breast cancer patients [32,33], suggesting a complex relationship between this novel serum biomarker and steroid hormones such as estrogen and progesterone.

Gene expression was also evaluated in response to EGF and gefitinib. The purpose of these studies was to explore the genomic pathways activated or inhibited downstream of EGFR in cells that are model of type I endometrial cancer and somewhat responsive to tyrosine kinase activation or inhibition versus those type II endometrial cancer that are resistant. The most striking observation is the apparent resistance of Hec50co cells to gefitinib at the transcriptional level: the pattern of gene expression was altered for fewer transcripts in response to gefitinib compared to the more robust response observed in Ishikawa H cells. These data are in agreement with our previous work indicating that gefitinib had little effect on Hec50co signaling pathways as assessed by phosphopeptide mapping [12] and analysis of cell cycle signaling events [13]. Together, these results combined with the genomic data presented here confirm that poorly differentiated Hec50co cells are resistant to gefitinib at multiple points downstream of EGFR signaling. We previously have linked gefitinib resistance in Hec50co cells with expression of the endogenous inhibitor of p53, MDM2, the phospho-activation of which is not fully blocked by gefitinib in this cell line [13]. Gene expression data from the two cell lines was further analyzed using Venn diagrams (Additional File 2, Figure S1). Alterations in the expression pattern of four differentially expressed genes were confirmed by qPCR. Interestingly, EGFR blockade modulates the downstream transcription activity of glucocorticoid receptor, predicting an anti-inflammatory and anti-proliferative effect in Ishikawa H cells.

Another interesting gene product, the dual specificity phosphastase (specific for ERK; DUSP6), was induced by EGF at 12 and 24 h (2.839 and 2.867 fold, respectively) and down-regulated by gefitinib treatment at 12 h and 24 h (0.288 and 0.381 fold, respectively) in Ishikawa H cells (Additional File 1, Table S1). These changes were confirmed by qPCR (Figure 5). Such changes in DUSP6 expression appear to form part of a negative feedback loop where EGF induces DUSP6, which then inhibits ERK signaling downstream of EGFR.

Two other gene products, sphingosine kinase 2 (SphK2) and EGR1, an early response transcription factor, were also differentially regulated in these two cell lines. SphK2 message was down-regulated by gefitinib at 12 h (0.445 fold) and induced by gefitinib at 24 h (2.172 fold) in Ishikawa H cells (Additional File 1, Table S1), indicating a potential biphasic effect of gefitinib on apoptosis (inhibited at 12 h, but induced at 24 h). EGR1, in contrast, is a central target of EGFR signaling that is induced by receptor activation and inhibited by tyrosine kinase blockade (Figure 5). Further studies may be undertaken to determine whether inhibition of EGR1 expression by tyrosine kinase inhibitors correlates with clinical response in patients; here we identify EGR1 as a potential marker of gefitinib response that warrants future investigation.

The findings from the Venn diagrams and Ingenuity™ networks, depicted in Additional Files 2, 3, 4, 5, 6, 7, 8, 9 and 10 (Figures S1-S9), show the genes regulated at 12 and 24 h by EGF or gefitinib in both Ishikawa H and Hec50co cells in the context of their interacting pathways. Considering the expansive role of EGFR in cellular functions, the genes consistently regulated in these cell lines (Additional File 2, Figure S1) identifies a surprisingly small core set of transcripts which may have utility as markers of response to novel therapeutics targeting the EGF pathway in patients with endometrial cancer.

The gene networks demonstrate that Ishikawa H cells were robustly responsive to EGF at 12 h treatment, including the induction of the central gene discussed above, EGR1 (Additional File 3, Figure S2). EGR1 is functionally linked to other genes upregulated by EGF treatment at 12 h, including FOS, SMAD3, FOSL1, FGFR1, MMP1, ETS1, SERPINE1, DUSP6, etc., which constitute a growth-promoting pathway in response to EGF (Additional File 3, Figure S2); of this pathway, EGR1, SMAD3, FOSL1, and MMP1 were also upregulated at 24 h of EGF treatment (Additional File 4, Figure S3). Of interest, this growth promoting pathway is linked to an immune modulatory transcriptional response, with IL-1β, IL-8, TNFRF21, and TNFRSF10A all induced. It is, perhaps, not surprising that proliferation and inflammation pathways are linked responses to EGF in endometrial carcinoma-derived cells, as has been observed in other models of cancer including hepatocellular carcinoma [34].

Consistent with this observation that an EGR1-containing pathway was upregulated by EGF treatment of Ishikawa H cells, treatment with the EGFR inhibitor gefitinib dampened EGR1 expression in Ishikawa H cells at 12 h (Additional File 5, Figure S4) and 24 h (Additional File 6, Figure S5), along with concomitant downregulation of expression of downstream genes such as FOSL1. This core group of EGF-responsive genes was regulated in the opposite manner by gefitinib. DUSP4 expression was also inhibited by gefitinib, and may constitute another marker gene of gefitinib response.

EGF/gefitinib-mediated transcriptional regulation in the type II EC cell line Hec50co was generally blunted but shared similarities with those observed in Ishikawa H cells, as well as some surprising differences. EGF-mediated EGR1 expression was upregulated at 12 h (Additional File 7, Figure S6), but not by 24 h (Additional File 8, Figure S7) in Hec50co cells; gefitinib treatment failed to inhibit EGR1 expression (Additional File 7, Figure S6 and Additional File 8, Figure S7, respectively). Surprisingly, EGF treatment caused a down-regulation of FGFR2 expression in Hec50co cells at both 12 h and 24 h.

At 12 h treatment (Additional File 9, Figure S8), gefitinib induced the expression of HDAC5, a growth inhibitory tumor suppressor and pro-apoptotic factor [35], in Hec50co cells, while regulators of cell migration and morphogenesis such as GIT1 and CALD1 were down-regulated by gefitinib. Also at 12 h of gefitinib treatment, APBA2 and RSP2, previously found to be highly expressed in endometrial carcinomas [36] and prostate cancer [37], respectively, were inhibited. However, by 24 h treatment (Additional File 10, Figure S9), Hec50co cells demonstrated a transcriptional response that may be compensatory to the anti-proliferative effects of gefitinib in that some genes associated with malignant transformation and tumor progression were induced. For example, ENC-1, often over-expressed in some types of leukemia [38], KLF12, a transcription factor associated with progression of gastric cancer [39], and CDC42EP2, a small Rho GTPase binding protein involved in hypoxia-induced angiogenesis [40], were induced by gefitinib at 24 h treatment. The induction of SMAD5 by gefitinib is also intriguing. SMAD5 has been characterized alternatively as a tumor suppressor or growth promoter, depending on cell context, so its specific function in response to gefitinib will require further study to fully understand.

In summary, the Ingenuity™ networks suggest several interesting findings. Ishikawa H cells are relatively responsive at the level of transcription to the growth-promoting effects of EGF and the growth inhibitory effect of gefitinib, as exemplified by the induction or inhibition of expression of a set of core genes including EGR1. Hec50co cells, however, show less of a proliferative response to EGF and an anti-proliferative response to gefitinib, with possible compensatory signaling apparent at 24 h treatment.

## Conclusions

In summary, to the extent that Ishikawa H cells model type I tumors, it is predicted that type I but not type II endometrial cancers have the capacity to respond therapeutically to EGFR-targeted tyrosine kinase inhibitors. Yet sequence analysis of hot spots within the tyrosine kinase domain of the EGFR in both type I and type II-derived cell lines revealed no mutations that might result in such differential sensitivity. Hec50co cells do, however, express lower levels of both sEGFR and EGFR, and such differences are consistent with previous reports indicating that serum sEGFR levels may be a useful indicator of responsiveness to gefitinib in other cancers, and also with the observation that serum sEGFR is a useful predictor of overall survival in endometrial cancer patients. In addition, our results suggest that type II endometrial tumors may be more resistant to EGFR-targeted therapies, at least in part, because they lack a genomic response that includes the modulation of the gene products described above in type I-derived endometrial cancer cells. If this hypothesis is correct, the changes in gene expression patterns observed here also may reveal potential biomarkers that warrant further investigation as surrogate biomarkers of responsiveness or resistance to gefitinib.

## Competing interests

The authors declare that they have no competing interests.

## Authors' contributions

All authors read and approved the final manuscript. LA designed and conducted the studies, carried out corresponding data analyses (other than where indicated below), and drafted the manuscript. GP analyzed the microarray data. MM performed the microarray analysis. JAW participated in study design, carried out the immunoassays, and helped to draft the manuscript. NJM participated in study design, and helped to draft the manuscript. KKL conceived of the study, oversaw its design and the experimental plan and completed the manuscript.

## Supplementary Material

Additional file 1 **Table S1**. List of genes regulated by EGF and gefitinib at 12 h and 24 h in Ishikawa H cells versus Hec50co cells. Click here for file

Additional file 2 **Figure S1**. Venn diagrams depicting the commonly and differentially regulated transcripts by EGF and gefitinib (Iressa) in Ishikawa H and Hec50co cells. Click here for file

Additional file 3 **Figure S2**. Ingenuity™ network depicting the transcriptional pathway most highly regulated in Ishikawa H cells treated with EGF for 12 h. Click here for file

Additional file 4 **Figure S3**. Ingenuity™ network depicting the transcriptional pathway most highly regulated in Ishikawa H cells treated with EGF for 24 h. Click here for file

Additional file 5 **Figure S4**. Ingenuity™ network depicting the transcriptional pathway most highly regulated in Ishikawa H cells treated with gefitinib (Iressa) for 12 h. Click here for file

Additional file 6 **Figure S5**. Ingenuity™ network depicting the transcriptional pathway most highly regulated in Ishikawa H cells treated with gefitinib (Iressa) for 24 h. Click here for file

Additional file 7 **Figure S6**. Ingenuity™ network depicting the transcriptional pathway most highly regulated in Hec50co cells treated with EGF for 12 h. Click here for file

Additional file 8 **Figure S7**. Ingenuity™ network depicting the transcriptional pathway most highly regulated in Hec50co cells treated with EGF for 24 h. Click here for file

Additional file 9 **Figure S8**. Ingenuity™ network depicting the transcriptional pathway most highly regulated in Hec50co cells treated with gefitinib (Iressa) for 12 h. Click here for file

Additional file 10 **Figure S9**. Ingenuity™ network depicting the transcriptional pathway most highly regulated in Hec50co cells treated with gefitinib (Iressa) for 24 h. Click here for file
