# Supplementary material for: EGFR isoforms and gene regulation in human endometrial cancer cells
Source: Mol Cancer. 2010 Jun 25;9:166. doi: 10.1186/1476-4598-9-166 (PMC2907331; doi:10.1186/1476-4598-9-166)
Supplement: Additional file 1 — Table S1. List of genes regulated by EGF and gefitinib at 12 h and 24 h in Ishikawa H cells versus Hec50co cells. [file 1476-4598-9-166-S1.DOC]

**Table S1. Differential Gene Expression** **in Ishikawa H and Hec50co Cells.** This table shows the genes and their fold change in response to EGF and gefitinib treatment at 12h and 24h.

| **Core transcripts downstream of EGFR differentially expressed in response to EGF and gefitinib at 12h in Ishikawa H cells** | | | | |
| --- | --- | --- | --- | --- |
| **Probe** | **EGF** | **Gefitinib** | **Gene description** | **Gene** |
| 201694_s_at | 4.701 | 0.496 | early growth response 1 | EGR1 |
| 201325_s_at | 4.043 | 0.4 | epithelial membrane protein 1 | EMP1 |
| 227404_s_at | 3.365 | 0.312 | early growth response 1 | EGR1 |
| 204420_at | 3.358 | 0.435 | FOS-like antigen 1 | FOSL1 |
| 208893_s_at | 3.278 | 0.324 | dual specificity phosphatase 6 | DUSP6 |
| 211668_s_at | 2.982 | 0.487 | plasminogen activator, urokinase | PLAU |
| 229011_at | 2.912 | 0.488 | epithelial membrane protein 1 | EMP1 |
| 208891_at | 2.839 | 0.288 | dual specificity phosphatase 6 | DUSP6 |
| 204011_at | 2.601 | 0.28 | sprouty homolog 2 (Drosophila) | SPRY2 |
| 208892_s_at | 2.473 | 0.323 | dual specificity phosphatase 6 | DUSP6 |
| 221489_s_at | 2.196 | 0.338 | sprouty homolog 4 (Drosophila) | SPRY4 |
| 227647_at | 2.009 | 0.319 | potassium voltage-gated channel, Isk-related family, member 3 | KCNE3 |
|  |  |  |  |  |
| **Core transcripts downstream of EGFR differentially expressed in response to EGF and gefitinib at 24h in Ishikawa H cells** | | | | |
| **Probe** | **EGF** | **Gefitinib** | **Gene description** | **Gene** |
| 201324_at | 3.301 | 0.274 | epithelial membrane protein 1 | EMP1 |
| 201325_s_at | 3.118 | 0.27 | epithelial membrane protein 1 | EMP1 |
| 208891_at | 2.867 | 0.381 | dual specificity phosphatase 6 | DUSP6 |
| 227404_s_at | 2.554 | 0.386 | early growth response 1 | EGR1 |
| 204011_at | 2.471 | 0.291 | sprouty homolog 2 (Drosophila) | SPRY2 |
| 206884_s_at | 2.437 | 0.446 | sciellin | SCEL |
| 1554921_a_at | 2.418 | 0.193 | sciellin | SCEL |
| 203939_at | 2.404 | 0.409 | 5'-nucleotidase, ecto (CD73) | NT5E |
| 208893_s_at | 2.337 | 0.298 | dual specificity phosphatase 6 | DUSP6 |
| 208892_s_at | 2.267 | 0.368 | dual specificity phosphatase 6 | DUSP6 |
| 222923_s_at | 2.267 | 0.368 | potassium voltage-gated channel, Isk-related family, member 3 | KCNE3 |
|  |  |  |  |  |
| **No transcript met the criteria of being differentially regulated by EGF and gefitinib in Hec50co cells at 12h or 24 h** | | | | |
|  | | | |  |
| **Transcripts regulated by EGF treatment at both 12h & 24h in Ishikawa H cells** | | | | |
| **Probe** | **12 h** | **24 h** | **Gene description** | **Gene** |
| 201694_s_at | 4.701 | 3.051 | early growth response 1 | EGR1 |
| 232056_at | 4.468 | 3.56 | sciellin | SCEL |
| 204475_at | 4.115 | 5.977 | matrix metalloproteinase 1 (interstitial collagenase) | MMP1 |
| 201325_s_at | 4.043 | 3.118 | epithelial membrane protein 1 | EMP1 |
| 227475_at | 3.89 | 4.713 | forkhead box Q1 | FOXQ1 |
| 209457_at | 3.718 | 2.621 | dual specificity phosphatase 5 | DUSP5 |
| 202859_x_at | 3.71 | 2.081 | interleukin 8 | IL8 |
| 202627_s_at | 3.705 | 2.098 | serine (or cysteine) proteinase inhibitor, clade E (nexin, plasminogen activator inhibitor type 1), member 1 | SERPINE1 |
| 209803_s_at | 3.564 | 2.978 | tumor suppressing subtransferable candidate 3 | TSSC3 |
| 201324_at | 3.476 | 3.301 | epithelial membrane protein 1 | EMP1 |
| 223541_at | 3.37 | 4.411 | hyaluronan synthase 3 | HAS3 |
| 227404_s_at | 3.365 | 2.554 | early growth response 1 | EGR1 |
| 204420_at | 3.358 | 2.683 | FOS-like antigen 1 | FOSL1 |
| 208893_s_at | 3.278 | 2.337 | dual specificity phosphatase 6 | DUSP6 |
| 1554921_a_at | 3.193 | 2.418 | sciellin | SCEL |
| 206884_s_at | 3.032 | 2.437 | sciellin | SCEL |
| 205479_s_at | 3.014 | 2.546 | plasminogen activator, urokinase | PLAU |
| 211668_s_at | 2.982 | 3.053 | plasminogen activator, urokinase | PLAU |
| 229011_at | 2.912 | 4.065 | epithelial membrane protein 1 | EMP1 |
| 203234_at | 2.903 | 2.037 | uridine phosphorylase | UP |
| 208891_at | 2.839 | 2.867 | dual specificity phosphatase 6 | DUSP6 |
| 209277_at | 2.829 | 3.338 | tissue factor pathway inhibitor 2 | TFPI2 |
| 202241_at | 2.772 | 2.021 | phosphoprotein regulated by mitogenic pathways | C8FW |
| 209386_at | 2.721 | 4.558 | transmembrane 4 superfamily member 1 | TM4SF1 |
| 204602_at | 2.712 | 2.641 | dickkopf homolog 1 (Xenopus laevis) | DKK1 |
| 205266_at | 2.711 | 2.099 | leukemia inhibitory factor (cholinergic differentiation factor) | LIF |
| 213895_at | 2.652 | 3.074 | epithelial membrane protein 1 | EMP1 |
| 204011_at | 2.601 | 2.471 | sprouty homolog 2 (Drosophila) | SPRY2 |
| 208322_s_at | 2.56 | 2.857 | sialyltransferase 4A (beta-galactoside alpha-2,3-sialyltransferase) | SIAT4A |
| 208892_s_at | 2.473 | 2.267 | dual specificity phosphatase 6 | DUSP6 |
| 204363_at | 2.459 | 2.254 | coagulation factor III (thromboplastin, tissue factor) | F3 |
| 200665_s_at | 2.357 | 3.569 | secreted protein, acidic, cysteine-rich (osteonectin) | SPARC |
| 205289_at | 2.289 | 6.486 | bone morphogenetic protein 2 | BMP2 |
| 201631_s_at | 2.264 | 2.158 | immediate early response 3 | IER3 |
| 203510_at | 2.186 | 2.21 | met proto-oncogene (hepatocyte growth factor receptor) | MET |
| 213524_s_at | 2.168 | 3.348 | putative lymphocyte G0/G1 switch gene | G0S2 |
| 205397_x_at | 2.162 | 4.291 | MAD, mothers against decapentaplegic homolog 3 (Drosophila) | MADH3 |
| 205097_at | 2.141 | 2.037 | solute carrier family 26 (sulfate transporter), member 2 | SLC26A2 |
| 217028_at | 2.038 | 2.437 | Homo sapiens CXCR4 gene encoding receptor CXCR4. | CXCR4 |
| 1552510_at | 0.496 | 0.442 | solute carrier family 34 (sodium phosphate), member 3 | SLC34A3 |
| 224482_s_at | 0.481 | 0.443 | rab11-family interacting protein 4 | RAB11-FIP4 |
| 209547_s_at | 0.472 | 3.639 | splicing factor 4 | SF4 |
| 206517_at | 0.399 | 0.428 | cadherin 16, KSP-cadherin | CDH16 |
| 212935_at | 0.372 | 0.186 | MCF.2 cell line derived transforming sequence-like | MCF2L |
|  |  |  |  |  |
| **Transcripts regulated by gefitinib treatment at both 12h & 24h in Ishikawa H cells** | | | | |
| **Probe** | **12 h** | **24 h** | **Gene description** | **Gene** |
| 235050_at | 2.068 | 2.112 | solute carrier family 2 (facilitated glucose transporter), member 12 | SLC2A12 |
| 210735_s_at | 0.47 | 0.256 | carbonic anhydrase XII | CA12 |
| 212558_at | 0.462 | 0.428 | ganglioside-induced differentiation-associated protein 1-like 1 | GDAP1L1 |
| 203662_s_at | 0.458 | 0.389 | tropomodulin 1 | TMOD1 |
| 203963_at | 0.448 | 0.29 | carbonic anhydrase XII | CA12 |
| 209857_s_at | 0.445 | 2.172 | sphingosine kinase 2 | SPHK2 |
| 201796_s_at | 0.424 | 0.471 | valyl-tRNA synthetase 2 | VARS2 |
| 201325_s_at | 0.4 | 0.27 | epithelial membrane protein 1 | EMP1 |
| 231315_at | 0.392 | 0.114 | thyroid transcription factor 1 | TITF1 |
| 212879_x_at | 0.373 | 2.295 | protein inhibitor of activated STAT protein PIASy | PIASY |
| 227647_at | 0.368 | 0.319 | potassium voltage-gated channel, Isk-related family, member 3 | KCNE3 |
| 222923_s_at | 0.355 | 0.45 | potassium voltage-gated channel, Isk-related family, member 3 | KCNE3 |
| 221489_s_at | 0.338 | 0.404 | sprouty homolog 4 (Drosophila) | SPRY4 |
| 204015_s_at | 0.332 | 0.384 | dual specificity phosphatase 4 | DUSP4 |
| 208893_s_at | 0.324 | 0.298 | dual specificity phosphatase 6 | DUSP6 |
| 208892_s_at | 0.323 | 0.368 | dual specificity phosphatase 6 | DUSP6 |
| 227404_s_at | 0.312 | 0.386 | early growth response 1 | EGR1 |
| 225503_at | 0.294 | 0.398 | dehydrogenase/reductase (SDR family) X chromosome | DHRSX |
| 203349_s_at | 0.293 | 0.312 | ets variant gene 5 (ets-related molecule) | ETV5 |
| 208891_at | 0.288 | 0.381 | dual specificity phosphatase 6 | DUSP6 |
| 204933_s_at | 0.281 | 0.479 | tumor necrosis factor receptor superfamily, member 11b (osteoprotegerin) | TNFRSF11B |
| 204011_at | 0.28 | 0.291 | sprouty homolog 2 (Drosophila) | SPRY2 |
| 216375_s_at | 0.273 | 0.367 | ets variant gene 5 (ets-related molecule) | ETV5 |
| 222922_at | 0.27 | 0.245 | potassium voltage-gated channel, Isk-related family, member 3 | KCNE3 |
| 203348_s_at | 0.236 | 0.32 | ets variant gene 5 (ets-related molecule) | ETV5 |
|  |  |  |  |  |
| **Genes regulated by EGF treatment at both 12h & 24h in Hec50co cells** | | | | |
| **Probe** | **12 h** | **24 h** | **Gene description** | **Gene** |
| 203638_s_at | 0.386 | 0.467 | fibroblast growth factor receptor 2 | FGFR2 |
| 204595_s_at | 2.293 | 2.204 | stanniocalcin 1 | STC1 |
| 234307_s_at | 0.442 | 0.156 | kinesin family member 26A | KIF26A |
|  |  |  |  |  |
| **Genes regulated by gefitinib treatment at both 12h & 24h in Hec50co cells** | | | | |
| **Probe** | **12 h** | **24 h** | **Gene description** | **Gene** |
| 1568704_a_at | 0.459 | 0.496 | calcium homeostasis endoplasmic reticulum protein | CHERP |
|  | | | | |
| **Common genes in Ishikawa H and Hec50co cells responsive to EGF at 12h** | | | | |
| **Probe** | **Ishikawa H** | **Hec50co** | **Gene description** | **Gene** |
| 227404_s_at | 3.365 | 2.442 | early growth response 1 | EGR1 |
| 217929_s_at | 0.248 | 0.373 | polycystic kidney disease 1-like | PKD1-like |
|  |  |  |  |  |
|  |  |  |  |  |
| **Common genes in Ishikawa H and Hec50co cells responsive to EGF at 24h** | | | | |
| **Probe** | **Ishikawa H** | **Hec50co** | **Gene description** | **Gene** |
| 226435_at | 0.328 | 0.482 | papilin, proteoglycan-like sulfated glycoprotein | PAPLN |
| 203638_s_at | 0.372 | 0.467 | fibroblast growth factor receptor 2 (bacteria-expressed kinase, keratinocyte growth factor receptor, craniofacial dysostosis 1, Crouzon syndrome, Pfeiffer syndrome, Jackson-Weiss syndrome) | FGFR2 |
| 206517_at | 0.428 | 0.46 | cadherin 16, KSP-cadherin | CDH16 |
| 203639_s_at | 0.267 | 0.208 | fibroblast growth factor receptor 2 (bacteria-expressed kinase, keratinocyte growth factor receptor, craniofacial dysostosis 1, Crouzon syndrome, Pfeiffer syndrome, Jackson-Weiss syndrome) | FGFR2 |
|  |  |  |  |  |
| **Common genes in Ishikawa H and Hec50co cells responsive to gefitinib at 12h** | | | | |
| **Probe** | **Ishikawa H** | **Hec50co** | **Gene description** | **Gene** |
| 202044_at | 0.483 | 2.73 | glucocorticoid receptor DNA binding factor 1 | GRLF1 |
|  |  |  |  |  |
| **Common genes in Ishikawa H and Hec50co cells responsive to gefitinib at 24h** | | | | |
| **Probe** | **Ishikawa H** | **Hec50co** | **Gene description** | **Gene** |
| 209857_s_at | 2.172 | 2.347 | sphingosine kinase 2 | SPHK2 |
| 212879_x_at | 2.295 | 2.06 | protein inhibitor of activated STAT protein PIASy | PIASY |
