# Supplementary material for: EGFR isoforms and gene regulation in human endometrial cancer cells
Source: Mol Cancer. 2010 Jun 25;9:166. doi: 10.1186/1476-4598-9-166 (PMC2907331; doi:10.1186/1476-4598-9-166)
Supplement: Additional file 2 — Figure S1. Venn diagrams depicting the commonly and differentially regulated transcripts by EGF and gefitinib (Iressa) in Ishikawa H and Hec50co cells. [file 1476-4598-9-166-S2.DOC]

**Figure S1. Venn diagram of genes commonly regulated in endometrial cancer cells by EGF and gefitinib treatment.** Left circle indicates genes regulated at 12h treatment, right circle indicates genes regulated at 24h treatment, bottom circle indicates genes not regulated by treatment. Overlap between left and right circle indicates genes commonly regulates at 12h and 24h. Panel A. Ishikawa H cells treated with EGF. Panel B. Ishikawa H cells treated with gefitinib. Panel C. Hec50co cells treated with EGF. Panel D. Hec50co cells treated with gefitinib. **
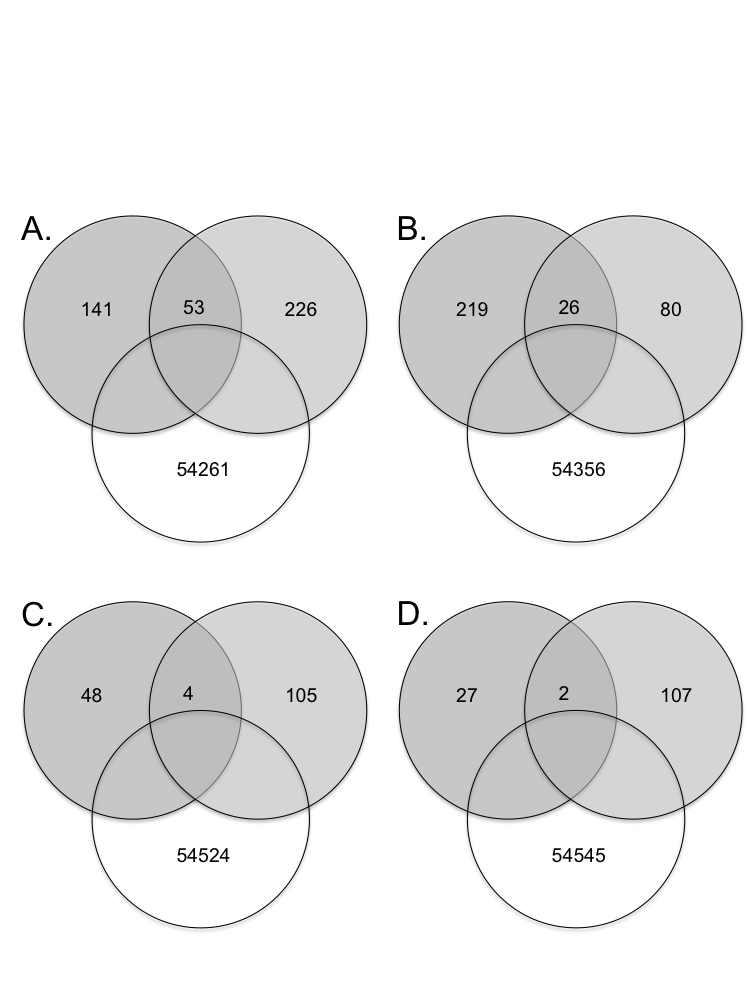
**
