# Supplementary material for: EGFR isoforms and gene regulation in human endometrial cancer cells
Source: Mol Cancer. 2010 Jun 25;9:166. doi: 10.1186/1476-4598-9-166 (PMC2907331; doi:10.1186/1476-4598-9-166)

**Figure S2. Pathway analysis of Ishikawa H cells treated with EGF for 12h.** This network demonstrates the interacting pathway most perturbed in Ishikawa H cells treated with EGF for 12h vs. vehicle treated cells. Red and green highlight indicate transcripts which are upregulated or down-regulated, respectively. This network demonstrates the interactions between the factors based upon published data from the sum of scientific publications reviewed.
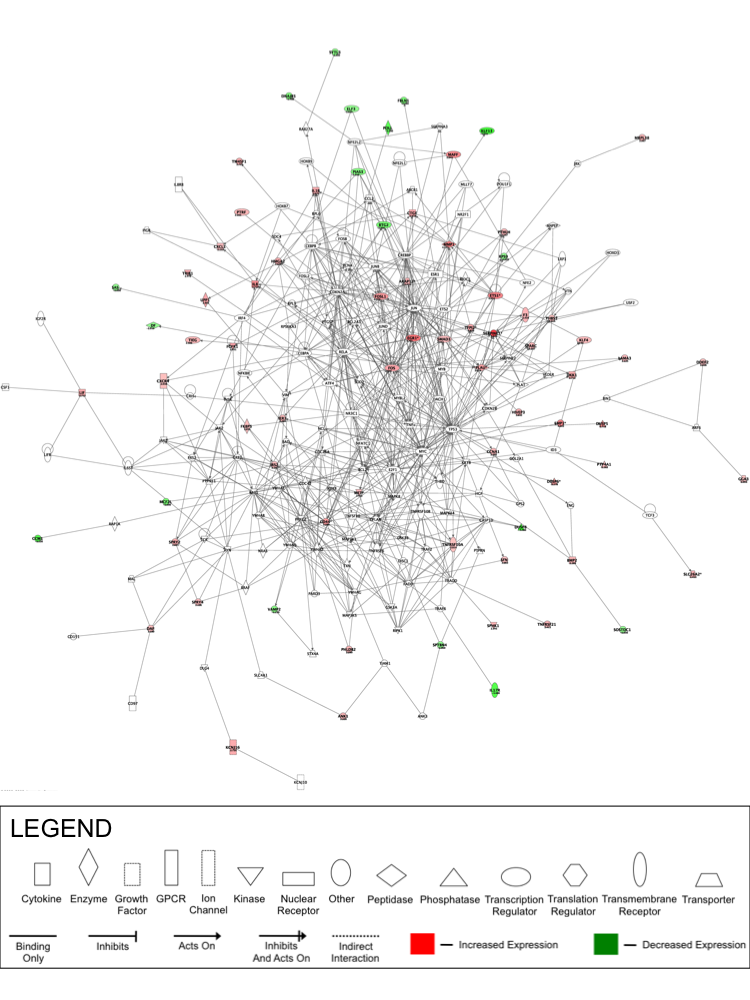

Supplement: Additional file 3 — Figure S2. Ingenuity™ network depicting the transcriptional pathway most highly regulated in Ishikawa H cells treated with EGF for 12 h. [file 1476-4598-9-166-S3.DOC]
