# Supplementary material for: EGFR isoforms and gene regulation in human endometrial cancer cells
Source: Mol Cancer. 2010 Jun 25;9:166. doi: 10.1186/1476-4598-9-166 (PMC2907331; doi:10.1186/1476-4598-9-166)
Supplement: Additional file 4 — Figure S3. Ingenuity™ network depicting the transcriptional pathway most highly regulated in Ishikawa H cells treated with EGF for 24 h. [file 1476-4598-9-166-S4.DOC]

**

**

**Figure S3. Pathway analysis of Ishikawa H cells treated with EGF for 24h.** This network describes the most significantly regulated pathways in Ishikawa H cells after 24h treatment with EGF.
