# Supplementary material for: EGFR isoforms and gene regulation in human endometrial cancer cells
Source: Mol Cancer. 2010 Jun 25;9:166. doi: 10.1186/1476-4598-9-166 (PMC2907331; doi:10.1186/1476-4598-9-166)
Supplement: Additional file 8 — Figure S7. Ingenuity™ network depicting the transcriptional pathway most highly regulated in Hec50co cells treated with EGF for 24 h. [file 1476-4598-9-166-S8.DOC]

**Figure S7. Pathway analysis of Hec50co cells treated with EGF for**

**24h.** This network describes the most significantly regulated pathways in Hec50co cells after 24h EGF treatment. **
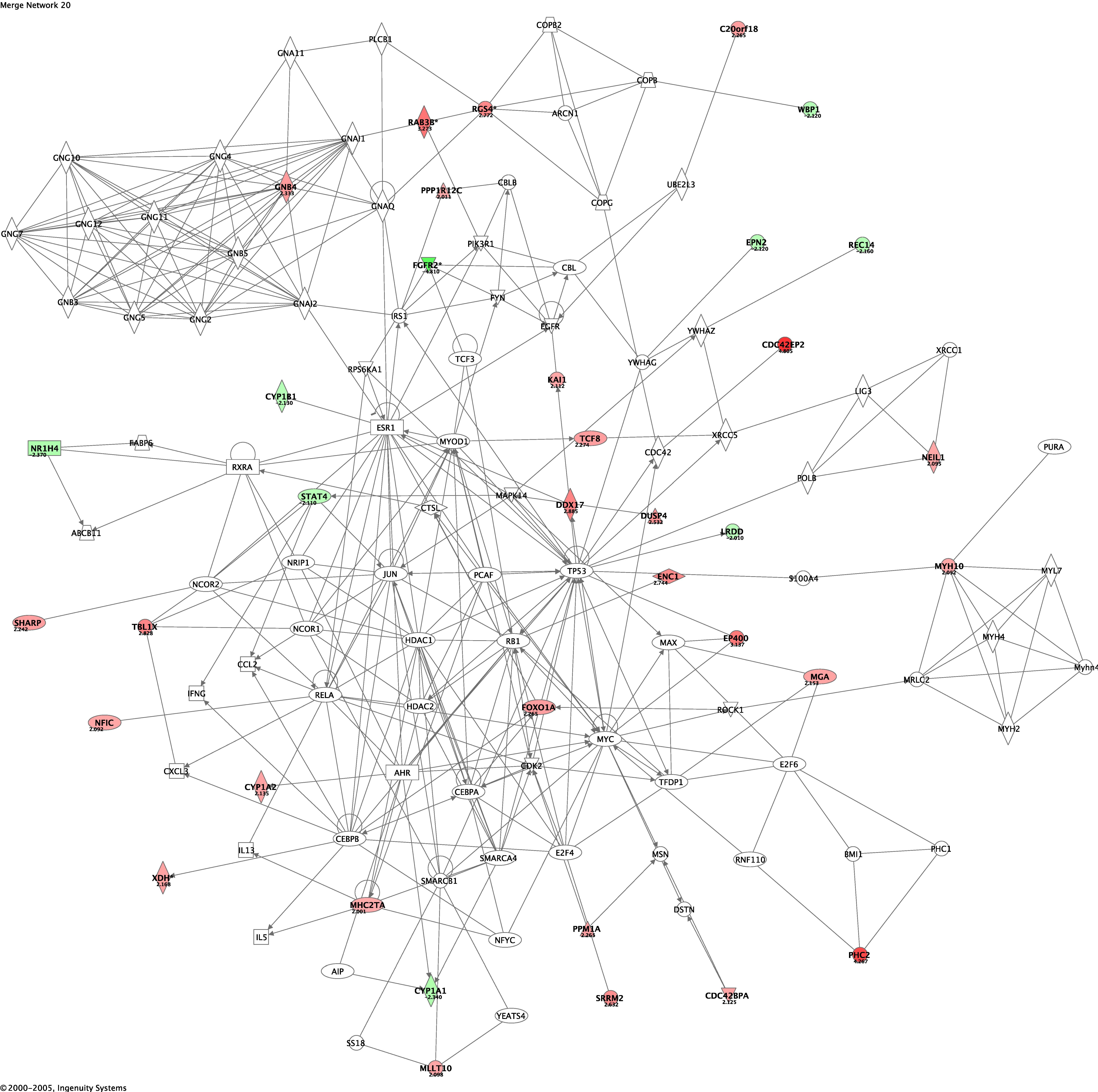
**
