# Supplementary material for: EGFR isoforms and gene regulation in human endometrial cancer cells
Source: Mol Cancer. 2010 Jun 25;9:166. doi: 10.1186/1476-4598-9-166 (PMC2907331; doi:10.1186/1476-4598-9-166)
Supplement: Additional file 9 — Figure S8. Ingenuity™ network depicting the transcriptional pathway most highly regulated in Hec50co cells treated with gefitinib (Iressa) for 12 h. [file 1476-4598-9-166-S9.DOC]

**Figure S8. Pathway analysis of Hec50co cells treated with gefitinib for 12h.** This network describes the most significantly regulated pathways in Hec50co cells after 12h treatment with gefitinib.

**
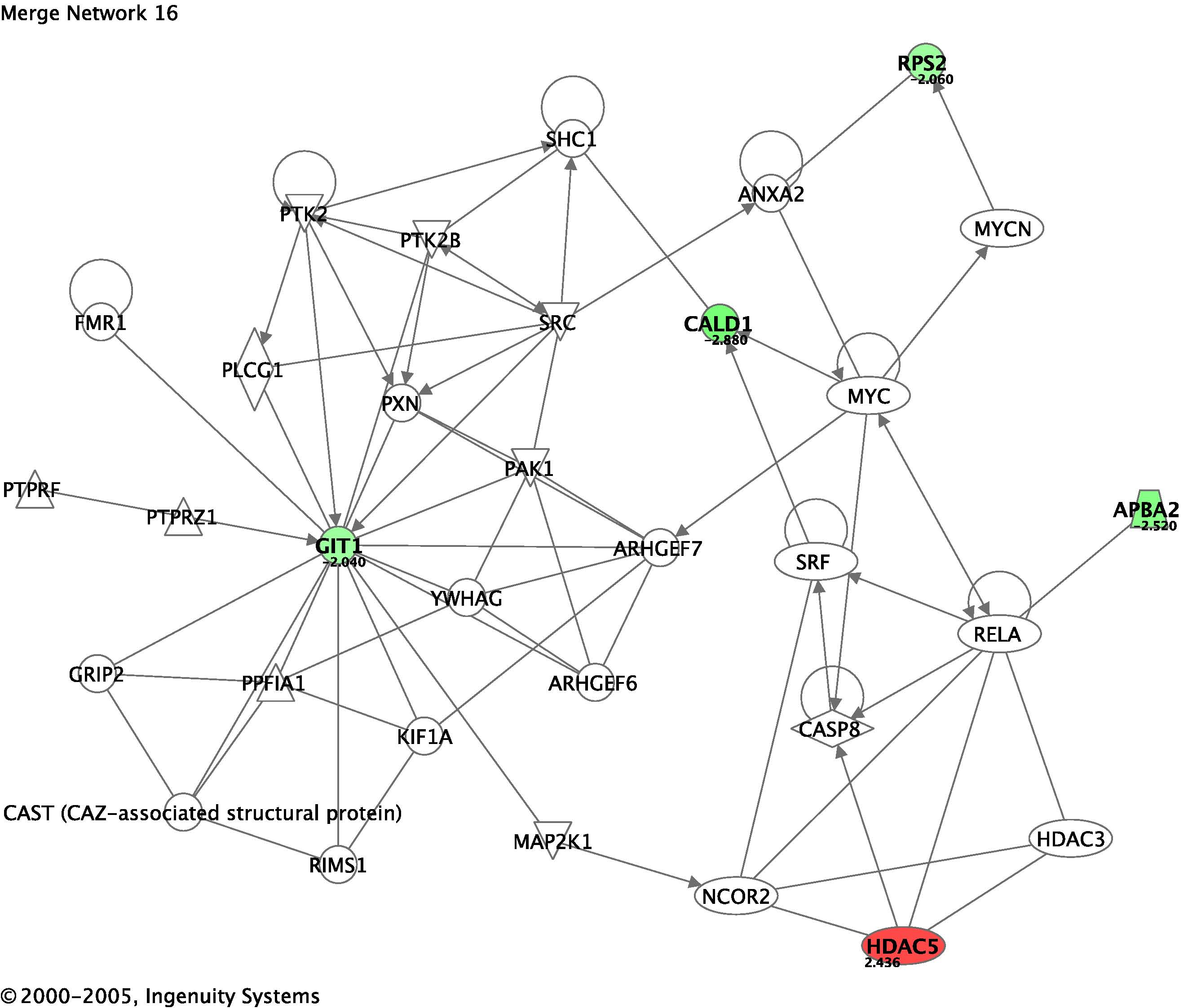
**
